# FBXO34 Regulates the G2/M Transition and Anaphase Entry in Meiotic Oocytes

**DOI:** 10.3389/fcell.2021.647103

**Published:** 2021-03-25

**Authors:** Bing-Wang Zhao, Si-Min Sun, Ke Xu, Yuan-Yuan Li, Wen-Long Lei, Li Li, Sai-Li Liu, Ying-Chun Ouyang, Qing-Yuan Sun, Zhen-Bo Wang

**Affiliations:** ^1^State Key Laboratory of Stem Cell and Reproductive Biology, Institute of Zoology, Chinese Academy of Sciences, Beijing, China; ^2^University of Chinese Academy of Sciences, Beijing, China; ^3^Fertility Preservation Lab, Reproductive Medicine Center, Guangdong Second Provincial General Hospital, Guangzhou, China; ^4^Institute for Stem Cell and Regenerative Medicine, Chinese Academy of Sciences, Beijing, China; ^5^Beijing Institute for Stem Cell and Regenerative Medicine, Beijing, China

**Keywords:** oocyte maturation, FBXO34, MPF, G2/M transition, SAC, anaphase entry

## Abstract

There are two important events in oocyte meiotic maturation, the G2/M transition and metaphase I progression. Thousands of proteins participate in regulating oocyte maturation, which highlights the importance of the ubiquitin proteasome system (UPS) in regulating protein synthesis and degradation. Skp1–Cullin–F-box (SCF) complexes, as the best characterized ubiquitin E3 ligases in the UPS, specifically recognize their substrates. F-box proteins, as the variable adaptors of SCF, can bind substrates specifically. Little is known about the functions of the F-box proteins in oocyte maturation. In this study, we found that depletion of FBXO34, an F-box protein, led to failure of oocyte meiotic resumption due to a low activity of MPF, and this phenotype could be rescued by exogenous overexpression of CCNB1. Strikingly, overexpression of FBXO34 promoted germinal vesicle breakdown (GVBD), but caused continuous activation of spindle assembly checkpoint (SAC) and MI arrest of oocytes. Here, we demonstrated that FBXO34 regulated both the G2/M transition and anaphase entry in meiotic oocytes.

## Introduction

The life of sexual reproduction originates from fertilized egg, the zygote of oocyte, and sperm after fertilization (Stitzel and Seydoux, 2007). Moreover, oocytes provide almost all cytoplasmic components for fertilization (Matzuk et al., 2002). Fully grown oocytes are arrested at prophase of the first meiosis, manifested by the germinal vesicle (GV) (Tripathi et al., 2010), and nuclear envelope breakdown termed germinal vesicle breakdown (GVBD) marks the resumption of meiosis. With the separation of homologous chromosomes, the oocytes accomplish the progression of the first meiosis, and after the first polar body extrusion, oocytes stagnate in the metaphase of the second meiosis (MII) (Jones, 2004; Tripathi et al., 2010; Fabritius et al., 2011), awaiting the arrival of the sperm and the occurrence of fertilization.

Similar to mitosis, the G2/M transition of meiosis, which is the resumption of meiosis, requires activation of maturation promoting factor (MPF) (Adhikari et al., 2012). There are two important components of MPF, the regulatory subunit CCNB1 (also known as cyclin B1) and the catalytic subunit CDK1 (also known as p34cdc2, CDC2, or cyclin-dependent kinase I) (Doree and Hunt, 2002). Wee1 protein kinase dephosphorylates CDK1 tyrosine residue at position 15 (Tyr-15), and then CDK1 is activated (Morgan, 1995; Mitra and Schultz, 1996). Because of continuous degradation of CCNB1 by anaphase-promoting complex/cyclosome (APC/C), the concentration of CCNB1 remains low before the resumption of meiosis (Marangos et al., 2007). Low levels of CCNB1 do not reach the threshold concentration for binding with CDK1, which is one of the reasons why MPF could not be activated and function (Reis et al., 2006). After the resumption of meiosis, the important event of oocyte is to initiate the metaphase/anaphase transition and complete the first meiosis (Tripathi et al., 2010; Fabritius et al., 2011). Spindle assembly checkpoint (SAC) plays an important role in monitoring the correct assembly of spindles and the accurate separation of chromosomes (Stegmeier et al., 2007). The spindle checkpoint proteins BubR1, Mad2, and Bub3 bind to CDC20, inhibiting the activity of APC and preventing segregation of chromosomes and anaphase entry (Reddy et al., 2007; Stegmeier et al., 2007; Li et al., 2009). Once all the chromosomes and spindles connect correctly and align on the equatorial plate, SAC begins to become inactivated and CDC20 activates APC. CCNB1 is rapidly degraded by APC/C, and the oocyte enters the anaphase (Reddy et al., 2007).

Thousands of proteins participate in the regulation of oocyte mature progress (Zhang et al., 2009). But at different developmental stages of oocytes, the protein compositions are different, correlating with oocyte characteristics (Wang et al., 2010). Thus, proteins are precisely translated and assembled before participating in life activities and then degraded in an orderly manner when no longer needed. The ubiquitin proteasome system (UPS) via posttranslational modifications regulates kinds of cellular processes, such as development and differentiation of cell, apoptosis, and cell cycle (Ciechanover, 1994; Hochstrasser, 1995). The UPS includes the activating E1 enzymes and the conjugating E2 enzymes, as well as the ligase E3 (Hershko et al., 2000; Pickart, 2004). The specificity of the UPS is monitored by E3 ligases, containing the major representative Skp1–Cullin–F-box (SCF) protein complex. The SCF complex consists of S-phase kinase–related protein 1 (SKP1), the backbone protein Cullin1, and a variable F-box protein (Cardozo and Pagano, 2004). Moreover, a recent research suggested that SCF ligases were necessary for oocytes maturation, expansion of cumulus cell, and fertilization (Kinterova et al., 2019). F-box proteins are a class of proteins that contain the F-box motif, which constitute the key component of the SCF and mainly mediate substrate recognition and substrate recruitment (Kipreos and Pagano, 2000). Moreover, F-box protein family also has many functions independent of SCF. Previous researches have revealed lots of functions of F-box proteins. High expression and phosphorylation of FBXO28 strongly and independently predict poor outcome in human breast cancer (Cepeda et al., 2013). FBXO32 impaired SK2, causing occurrence of diabetes mellitus (Ling et al., 2019). In addition, F-box proteins are also significantly important in germ cell development and maturation. FBXO43 (also known as Emi2) played a key role in spermatocytes at early diplotene of prophase I (Gopinathan et al., 2017) and inhibited the binding of Ube2S to APC/C, mediating meiotic MII arrest (Sako et al., 2014). FBXO30-deleted oocytes were arrested at MI stage, due to chromosome overcondensed and failure of separation (Jin et al., 2019). As a member of F-box proteins, FBXO34 is highly expressed in the testis and ovary of adult mice, but the functions of FBXO34 have hardly any reports.

Here, we found that depletion of FBXO34 led to the significant decrease of MPF activity and failure of GVBD. When FBXO34 was overexpressed, it was extremely difficult for oocytes to emit the first polar body and reach to MII stage. To deeply explore the mechanisms of the MI arrest, we performed the live-cell imaging experiments. Surprisingly, the homologous chromosomes did not separate, and the spindle of MI stage did not migrate to the cortex. Chromosome spreading experiments showed that FBXO34-overexpressed oocytes had a continuous activation of SAC. In our study, we revealed the significant function of FBXO34 protein in regulating the maturation of oocytes.

## Results

### Subcellular Localization of FBXO34 During Oocyte Meiotic Maturation

**FIGURE 1:**
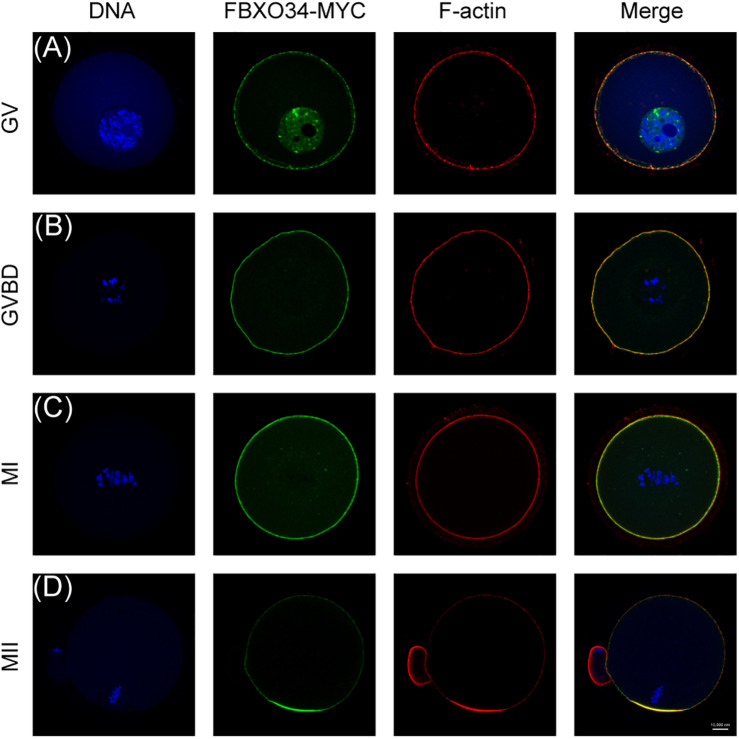
Subcellular localization of FBXO34 in oocyte meiotic stages. **(A–D)** Oocytes at GV, GVBD, MI, and MII stages were collected for immunofluorescent analysis for FBXO34 (green), DNA (blue), and F-actin (red). Scale bar, 10 μm.

We first checked the subcellular localization of FBXO34 during oocyte meiotic maturation. We collected mouse oocytes and microinjected them with FBXO34-MYC mRNAs and then cultured the oocytes to different stages. FBXO34 was mainly localized at the nucleus and colocalized with F-actin in the membrane of oocytes at GV stage (Figure 1A). The signal of FBXO34 was colocalized with F-actin in the membrane of oocytes at GVBD and MI stages (Figures 1B,C). At MII stage, it was concentrated in the cortex, overlapping the actin cap (Figure 1D).

### Depletion of FBXO34 Reduced MPF Activity and Caused Failure of Resumption of Meiosis

**FIGURE 2:**
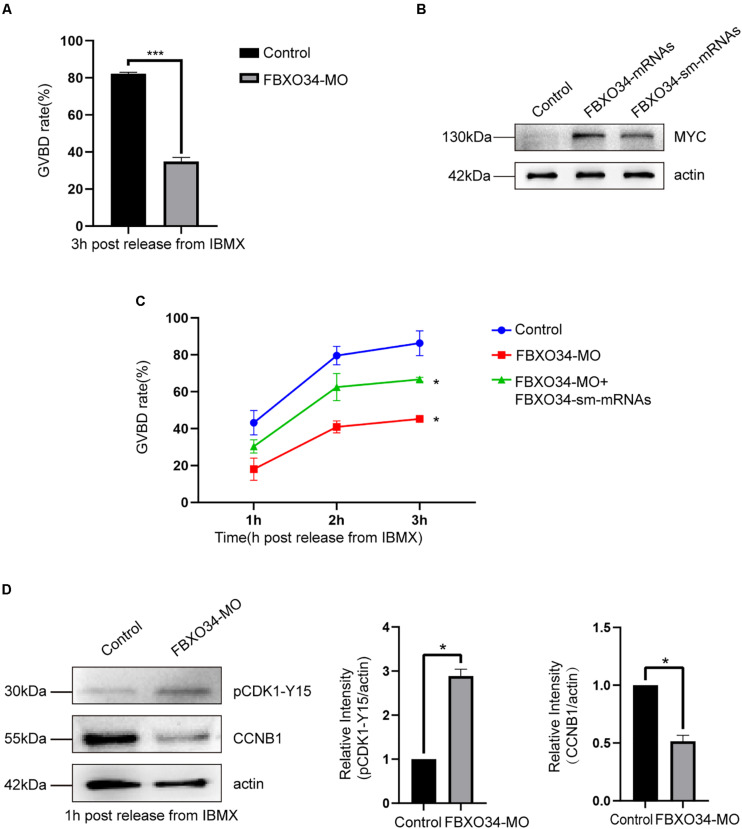
Depletion of FBXO34 impaired the resumption of meiosis and activity of MPF. **(A)** GVBD rates of control group and FBXO34-MO group at 3 h following release from IBMX inhibition. **(B)** Western blot of FBXO34-MYC and actin in control, FBXO34 mRNA–injected, and FBXO34-sm mRNA–injected oocytes (100 oocytes per sample). The molecular weight of FBXO34-MYC is 130 kDa; the molecular weight of actin is 42 kDa. **(C)** After oocytes were released from IBMX inhibition, GVBD rates of oocytes at 1, 2, and 3 h for control group; FBXO34-MO group; and FBXO34-MO+FBXO34-sm mRNA group were summarized. **(D)** After oocytes were released from IBMX inhibition, Western blot of the phosphorylation level of Tyr15 of Cdk1 (pCdk1-Y15), CCNB1, and actin in the control group and the FBXO34-MO group were presented (180 oocytes per sample). The molecular weight of pCDK1-Y15 is 30 kDa, the molecular weight of CCNB1 is 55 kDa, and the molecular weight of actin is 42 kDa. The relative intensity of immunoreactive bands was quantified by densitometry. Quantitative data were obtained from at least three independent repeats, and one repeat of each experiment contained at least 50 oocytes. The error bars of quantitative data represent the standard deviation (*P < 0.05, ***P < 0.005).

Morpholino (MO) was used to deplete FBXO34 in mouse oocytes. Interestingly, only 35% of FBXO34-depleted oocytes underwent GVBD by 3 h after being released from 3-isobutyl-1-methylxanthine (IBMX). It was inescapably clear that the percentage of oocytes passing through prophase I in the FBXO34-depleted group was lower than that in the control oocytes (34.9% ± 1.3% vs. 82.2% ± 0.5%; *P* < 0.05) (Figure 2A). To demonstrate the specificity of FBXO34-MO, we used depletion and rescue methods. Exogenous mRNAs with synonymous mutation of the ATG site prevent morpholino targeting and encode the same protein (Madakashira et al., 2017). Most importantly, the incidence of the prophase I arrest was significantly reduced by coinjection of the FBXO34-MO and synonymous mutant mRNAs (Figures 2B,C). These results suggested that FBXO34 was specifically and effectively depleted in our experiments, and these FBXO34-depleted oocytes showed failure of prophase I resumption, being kept arrest at the GV stage.

Given the above results, we further explored the underlying mechanisms. We tested the expression level of pCDK1 and CCNB1, two critical components of MPF (Jones, 2004). Normally, CDK1 become active by dephosphorylation at Tyr 15, and CCNB1 will be accumulated for GVBD. We found that the level of CDK1 phosphorylation has a significant increase in FBXO34-depleted oocytes comparing with the control group. Moreover, we examined that the level of CCNB1 in FBXO34-depleted oocytes was evidently lower than that in the control group (Figure 2D). We supposed that the reason for FBXO34-depleted oocytes failing to resume prophase I progression was caused by the low expression level of CCNB1 and lack of CDK1 dephosphorylation, which led to a low level of MPF activity.

### Overexpression of CCNB1 Could Rescue the Failed GVBD Caused by Depletion of FBXO34

**FIGURE 3:**
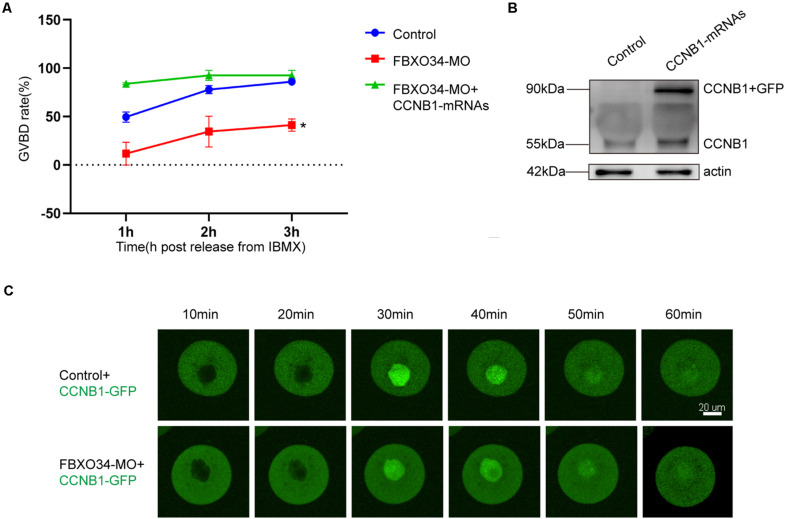
The addition of exogenous CCNB1 alleviated the impairment caused by FBXO34-MO. **(A)** GVBD rates of control, FBXO34-MO group, and FBXO34-MO+CCNB1-GFP mRNA group at 1, 2, and 3 h following release from IBMX. **(B)** Western blot of the CCNB1-GFP, endogenous CCNB1, and actin in the control group and the CCNB1-GFP mRNA group were presented (180 oocytes per sample). **(C)** The dynamics of CCNB1-GFP were shown by live-cell imaging in control oocytes and FBXO34-MO oocytes. Images of live cells were taken every 10 min from releasing to occurrence of GVBD. Scale bar, 20 μm. Quantitative data were obtained from at least three independent repeats, and one repeat of each experiment contained at least 50 oocytes. The error bars of quantitative data represent the standard deviation (*P < 0.05).

To explore the functions of CCNB1 in FBXO34-depleted oocytes, exogenous CCNB1-GFP mRNAs were injected, and the location of CCNB1 was traced in oocytes. CCNB1 distributed in the cytoplasm of oocytes at GV stage and then transferred to the nucleus before GVBD. Comparing with control oocytes, the nuclear entry of CCNB1 was not affected in FBXO34-depleted oocytes (Figure 3C and Supplementary Movies 1,2). However, overexpression of CCNB1 in FBXO34-depleted oocytes increased GVBD evidently after CCNB1-GFP mRNA injection (Figures 3A,B), which attested that FBXO34 might play an integrant role in stabilization of CCNB1.

### Overexpression of FBXO34 Promoted GVBD

**FIGURE 4:**
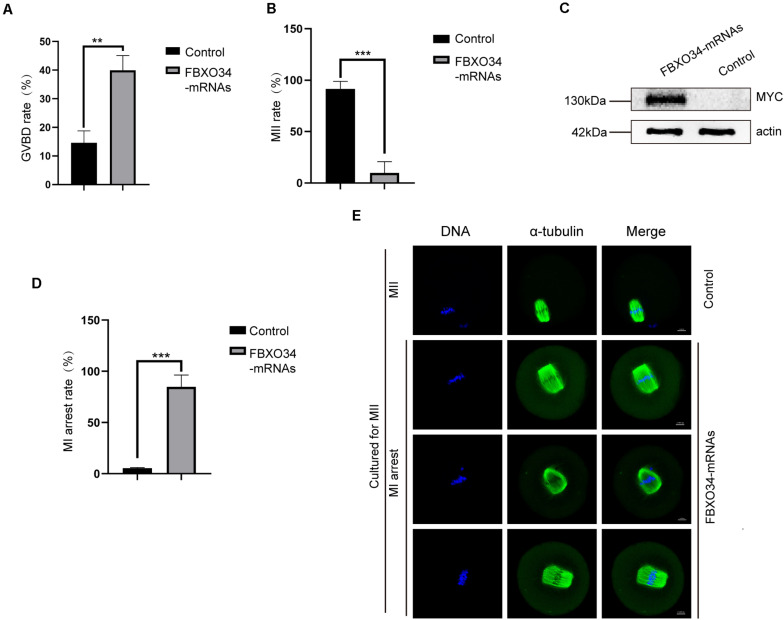
Effects of FBXO34 overexpression on resumption and progression of meiosis. **(A)** GVBD rates at 3 h after being released to M2 medium with 50 μM IBMX for control and FBXO34-overexpressed oocytes. **(B)** MII rates of control and FBXO34-overexpressed oocytes. **(C)** Western blot of FBXO34-MYC and actin in control, FBXO34 mRNA–injected oocytes (100 oocytes per sample). The molecular weight of FBXO34-MYC is 130 kDa, and the molecular weight of actin is 42 kDa. **(D)** The rates of MI arrest oocyte in control and FBXO34-overexpression groups. **(E)** Oocytes in the control and FBXO34-overexpression groups were cultured for 12 h followed with staining with α-tubulin (green) and DNA (blue). Scale bar, 10 μm. Quantitative data were obtained from at least three independent repeats, and one repeat of each experiment contained at least 50 oocytes. The error bars of quantitative data represent the standard deviation (**P < 0.01, ***P < 0.005).

To further gain insight into the roles of FBXO34 in the G2/M transition of oocytes, we performed the FBXO34 overexpression experiment. Oocytes microinjected with FBXO34 mRNAs were incubated in M2 medium with IBMX for 4 h, achieving overexpression of FBXO34 (Figure 4C). Then, the FBXO34-overexpressed oocytes were released to M2 medium with low concentration IBMX (50 μM). We found that FBXO34-overexpressd oocytes promoted the rate of GVBD comparing with control oocytes (Figure 4A). By knockdown and overexpression experiments, we deduced that FBXO34 was one of the indispensable proteins in the G2/M transition of oocytes.

### Overexpression of FBXO34 Blocked Anaphase Entry in Oocytes

Overexpression of FBXO34 promoted GVBD, and it affected succeeding development during oocyte maturation. When cultured for 12 h in M2 medium, almost all control oocytes (95.1% ± 1%; *P* < 0.05) emitted the first polar body. Contrarily, only very few FBXO34-overexpressed oocytes (8.0% ± 9.7%; *P* < 0.05) could exclude the first polar body (Figure 4B). We explored the reason that FBXO34-overexpressed oocytes were arrested at MI stage by immunofluorescent and live-cell imaging experiments. In the overexpressed oocytes, the two ends of the spindles were messy and irregular, the homologous chromosomes did not separate (Figure 4E), and those FBXO34-overexpressed oocytes were arrested at MI stage (Figure 4D).

**FIGURE 5:**
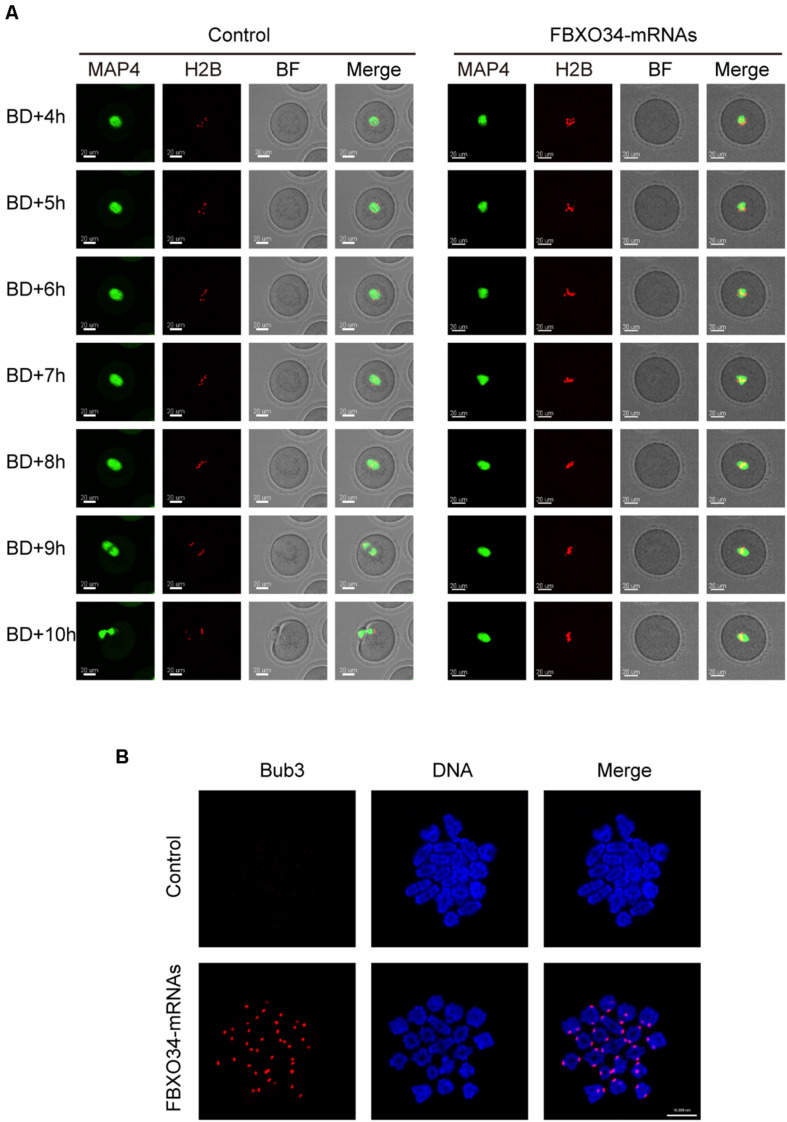
Overexpression of FBXO34 caused continuous activation of SAC and MI arrest. **(A)** Control and FBXO34-overexpressed oocytes injected with MAP4-eGFP and H2B-mCherry mRNAs were incubated for 4 h and released to M2 medium for live-cell imaging. The spindle was labeled with MAP4-eGFP, and the chromosomes were labeled with H2B-mCherry. Images of live cells were taken every 30 min from the start of releasing to the end of 14 h later. Scale bar, 20 μm. **(B)** After being incubated for 9.5 h, control and FBXO34-overexpressed oocytes were collected for chromosome spreading. Bub3 (red), DNA (blue). Scale bar, 10 μm.

In addition, we performed live-cell imaging experiment to analyze the dynamics of chromosomes and spindle of FBXO34-overexpressed oocytes. Spindle and chromosomes were labeled by MAP4-eGFP and H2B-mCherry, respectively (Li et al., 2020). In control oocytes, the spindles organized normally, the chromosomes aligned naturally, and the first polar body extruded on time (Figure 5A and Supplementary Movie 3). In contrast, although the chromosomes and the spindle are normal between the stages GVBD and MI in the FBXO34-overexpressed oocytes, the chromosomes and the spindle were at a standstill for the next step. The chromosomes did not separate, and the spindle did not migrate to the cortex (Figure 5A and Supplementary Movie 4).

Given that FBXO34-overexpressed oocytes were arrested at MI stage, we performed chromosome spreading to test the activity of SAC. Comparing with the control oocytes, the homologous chromosomes of FBXO34-overexpressed oocytes did not separate, and the signals of Bub3 were detected on chromosome kinetochores. However, we could not detect the signals of Bub3 in the control oocytes (Figure 5B). The result suggested that the SAC in FBXO34-overexpressed oocytes was still activated, which inhibited the chromosomes separation. The results above demonstrated that overexpression of FBXO34 led to failure of anaphase entry.

## Discussion

Mammalian oocytes mature with the involvement of thousands of proteins, many of which later follow the cytoplasm into the fertilized egg and participate in the subsequent life activities, such as zygotic gene activation and embryogenesis (Zhang et al., 2009; Wang et al., 2010). Therefore, the balance of proteins production and degradation is extremely important. The UPS plays a key role in protein degradation. In the UPS, F-box family proteins as the important component of SCF determined the substrate-selected specificity of E3 ligase (Weissman, 2001). In the human, F-box protein family contains almost 70 members (Skaar et al., 2009) that consist of cell-cycle regulators, DNA replication factors, cyclin-dependent kinase inhibitors, transcription factors, and so on (Kipreos and Pagano, 2000). In this study, we focused on the functions of FBXO34 in oocyte maturation. Interestingly, we found that FBXO34 played pivotal roles in oocyte maturation.

Oocytes are arrested at GV stage, which is the diplotene stage of the first meiotic prophase. After increased expression of CCNB1 and dephosphorylation of CDK1, the MPF was activated, and the resumption of meiosis occurred (Adhikari et al., 2012), marked as GVBD. In this study, we first used morpholino, a tool of targeting the start codon of FBXO34 transcript, to avoid original translation of FBXO34. We found that injection of FBXO34-MO caused failure of prophase I resumption. It was reported that FBXW7 as substrate recognition subunit played a key role in degradation of protein at meiotic prophase, especially the transition from pachytene to diplotene (Kisielnicka et al., 2018). It is well-known that MPF is an important regulator for the resumption of meiosis. Because of the low level of CCNB1 in FBXO34-depleted oocytes, we injected exogenous CCNB1-GFP mRNAs to rescue the resumption of meiosis in FBXO34-depleted oocytes. We found that CCNB1-GFP entered the nucleus, and GVBD occurred in FBXO34-depleted oocytes. It was verified that the failure of prophase I resumption in FBXO34-depleted oocytes was due to the low expression level of CCNB1. Similarly, a previous report mentioned that Emi1 (also known as FBXO5) was responsible for destruction of CCNB and inactivation of MPF (Marangos et al., 2007). We speculated that the decrease of CCNB1 level and the inactivation of MPF caused by depletion of FBXO34 might act in a similar way. Moreover, the SKP1 could directly bind CCNF (also known as cyclin F) through a novel structural motif and regulated proteolysis in G1/S and G2/M transitions (Bai et al., 1996). SCF-mediated ubiquitination was closely related to the regulation of meiosis resumption. In our work, we confirmed that the depletion of FBXO34 led to the low level of and inactivity of MPF, but the direct substrate of FBXO34 needs further investigation.

After the resumption of meiosis, the chromosomes attach to the spindle and align correctly, waiting for separation after the SAC deactivation (Dumont and Desai, 2012; Lara-Gonzalez et al., 2012; Stukenberg and Burke, 2015). In FBXO34-overexpressed oocytes, the chromosomes could not separate normally, stagnating in the MI stage. We found that the two ends of the spindle were messy and irregular, and the chromosomes did not separate. Furthermore, in contrast to control oocytes, the spindle of FBXO34-overexpressed oocytes did not migrate to the cortex. Continuous activation of the SAC leads to the failure of the oocyte meiotic progress (Lara-Gonzalez et al., 2012). Bub3, as a core member of SAC, monitors oocyte meiotic anaphase entry (Li et al., 2009). To further detect the reason of the MI arrest, we checked the activity of the SAC according to the signals of Bub3. The SAC kept continuous activation at 9.5-h culture in FBXO34-overexpressed oocytes. A previous study found that depletion of FBXO30 caused the overcondensed chromosomes and failure of chromosome segregation (Jin et al., 2019). In addition, as a member of F-box proteins, FBXO34 was a composition of the UPS, participating in regulation of protein production and degradation (Zhou and Howley, 1998; Galan and Peter, 1999). To some extent, overexpression of FBXO34 might increase the levels of ubiquitination, causing degradation of one or more proteins and impairment of meiosis progress. MPF plays a vital role during the meiosis I to meiosis II transition (Jones, 2004). FBXO34 depletion led to decrease of MPF activity and failure of GVBD. Thus, we speculated that overexpression of FBXO34 might impair the transition from meiosis I to meiosis II by regulating MPF activity. In our work, we provided a new evidence of F-box family proteins regulating oocyte maturation.

In conclusion, our study revealed the importance of FBXO34 in mouse oocyte maturation. The balance of protein is pivotal for unidirectional progression of cell cycle (Wang et al., 2017), especially the ubiquitination-related proteins. We demonstrated that the FBXO34 played a vital role both in the G2/M transition and anaphase entry in meiotic oocytes.

## Materials and Methods

### Oocyte Collection and Culture

6–8 week-old ICR mice were fed and handled according to the policies of the ethics committee of the Institute of Zoology, Chinese Academy of Sciences. To maintain the collected oocytes at GV stage, we used M2 medium with 200 μM IBMX to culture them. Moreover, we used the inhibitory model using a low concentration of IBMX (50 μM) to perform the experiment “overexpression of FBXO34 promoted GVBD.” Oocytes were released for culturing in M2 medium to different stages, such as GVBD (2 h), MI (8 h), and MII (12 h). M2 medium covered with mineral oil was used to culture the oocytes in a humidified atmosphere of 5% CO_2_ at 37°C.

### Plasmid Construction

RNeasy micro purification kit (Qiagen) was used to extract total RNA from 200 mouse GV oocytes as a sample, and then cDNA synthesis kit (Takara) and poly(dT) primers were used to generate the first strand cDNA. The full length of FBXO34 CDS was amplified by the polymerase chain reaction (PCR) method. The PCR products and Myc tag plasmid were, respectively, digested using *Fse*I and *Asc*I (New England Biolabs, Inc.), and then FBXO34 CDS and Myc plasmid were linked. The fusion plasmid was transfected into DH5α competent cells (Transgene Biotech). KOD-Plus-Mutagenesis Kit (Toyobo) was used to make a synonymous mutation of FBXO34 plasmid. CCNB1-GFP plasmid, MAP4-eGFP plasmid, and H2B-mCherry plasmid were acquired from plasmid bank in our laboratory.

### Microinjection of Morpholino and mRNAs

We used the Nikon operating system to achieve microinjection and complete within 30 min. Each oocyte was treated with 5–10 pl of MO or mRNAs. FBXO34-MO (5′-CGCTCGCCCCGAAACCCATTTGTTG-3′) (Gene Tools) was diluted with nuclease-free water to knockdown FBXO34 in mouse oocytes by microinjecting, and the same nuclease-free water was used for the control. mRNAs were produced and capped by the mMessage mMachine (Ambion), added the tail with poly(A) polymerase Tailing kit (Epicenter, AP-31220) and purified with an RNeasy cleanup kit (Qiagen). FBXO34 mRNAs and FBXO34-sm mRNAs sequences of the ATG site are as follows: FBXO34 mRNAs (5′-ATGGGTTTCGGGGCGAGCGTT-3′), FBXO34-sm mRNAs (5′-ATGGTTTTGGAGGCAAGCGTT-3′). For overexpression and localization experiments, oocytes were microinjected with 500 ng to 1 μg/μL and 50 ng to 200 ng/μL mRNAs into the cytoplasm of GV oocytes, respectively.

### Antibodies

The antibodies were as follows: anti-CCNB1 antibody (Santa Cruz Biotechnology; Cat#: sc-245; 1:2,000 for Western blot), anti-pCDK1 (Tyr 15) antibody (Santa Cruz Biotechnology; Cat#: sc-12340-R; 1:1,000 for Western blot), anti–β-actin antibody (Santa Cruz Biotechnology; Cat#: sc-8432; 1:3,000 for Western blot), anti-MYC antibody (Sigma-Aldrich; Cat#: M4439; 1:3,000 for Western blot), anti-MYC antibody (Thermo Fisher; Cat#: R953-25; 1:200 for immunofluorescent analysis), anti–α-tubulin–FITC antibody (Sigma-Aldrich; Cat#: F2168; 1:800 for immunofluorescent analysis), and Alexa Fluor^TM^ 546 Phalloidin (Thermo Fisher; Cat#: A22283; 1:200 for immunofluorescent analysis).

### Western Blot and Immunofluorescent Analysis

Western blot experiments were performed to analyze the expression level of proteins. Two hundred mouse oocytes as a sample were collected in 7 μL 1.5 × sodium dodecyl sulfate (SDS) buffer containing protease inhibitor and immediately boiled for 5 min. After separating by SDS–polyacrylamide gel electrophoresis, the denatured proteins were transferred to polyvinylidene fluoride membrane. Next, TBST (Tris-buffered saline with 0.1% Tween 20) containing 5% bovine serum albumin (BSA) was used to block the membrane for 1–2 h at room temperature. When incubated with primary antibodies for 16 h in a 4°C environment, the membrane was washed in TBST for three times (10 min for each time) and then incubated with corresponding secondary antibodies for 1 h at 37°C. After three washes in TBST, the membrane was handled by the enhanced chemiluminescence detection system (Bio-Rad).

Immunofluorescent experiments were performed to analyze the localization of proteins. Paraformaldehyde (PFA) 4% and Triton X-100 0.5% were used to fix oocytes for 30 min and permeabilize oocytes for 25 min successively. Then, blocking buffer (1% BSA in PBS) was used to block oocytes for 1–2 h at room temperature. After primary antibodies were incubated for 16 h in a 4°C environment, oocytes were washed in washing buffer (0.1% Tween 20 and 0.01% Triton X-100 in PBS) for three times (5 min for each time). Next, oocytes were labeled with the appropriate fluorescent secondary antibodies for 1–2 h at room temperature. After three washes, 4’,6-diamidino-2-phenylindole (DAPI, Sigma-Aldrich) was used to stain the chromatin of oocytes for 15 min. Confocal laser-scanning microscope (Zeiss LSM 880) was used for observing the immunofluorescent signals.

### Confocal Time-Lapse Live Imaging

After a mini-cell incubator was equipped, confocal microscope imaging system (Andor dragonfly 200) was used to perform live-cell imaging experiment. The injected oocytes were incubated in M2 medium covered by mineral oil. According to our requirement, the fluorescence, the whole incubation time, and the spacing were adjusted to take images.

### Chromosome Spreading

Chromosome spreading was performed as follows (Hodges and Hunt, 2002): First, the zona pellucida of oocytes was removed by acid Tyrode’s solution (Sigma) at room temperature. After a fast wash in M2 medium, the oocytes were moved onto a clean glass slide that dropped with a solution (1% PFA 0.15% Triton X-100 and 3 mM dithiothreitol in water). Then, the oocytes were blocked with blocking buffer for 1–2 h at room temperature. Then the oocytes were incubated with primary antibodies for 16 h at 4°C and washed three times (5 min for each time) with washing buffer. After the appropriate fluorescent secondary antibodies were incubated for 1–2 h and washed three times, oocytes were stained with DAPI for 15 min. Confocal laser-scanning microscope (Zeiss LSM 880) was used for observing the immunofluorescent signals.

### Statistical Analysis

The Photoshop CS5 (Adobe) and Illustrator CC5 (Adobe) were used for analysis of the images. Quantitative analysis of each experiment (mean ± standard error of mean) was obtained from repeating at least three times and processed by Student *t* test using Prism8 (GraphPad Software) with *P* < 0.05 set for significance.

## Data Availability Statement

The original contributions presented in the study are included in the article/Supplementary Material, further inquiries can be directed to the corresponding authors.

## Ethics Statement

The animal study was reviewed and approved by the Ethics Committee of the Institute of Zoology, Chinese Academy of Sciences.

## Author Contributions

B-WZ, Q-YS, and Z-BW conceived and designed the project and analyzed the data. B-WZ, S-MS, KX, W-LL, and S-LL prepared and performed the research. Y-YL and Y-CO provided the technical support. B-WZ and Z-BW wrote the manuscript. All authors contributed to the article and approved the final version of this manuscript.

## Conflict of Interest

The authors declare that the research was conducted in the absence of any commercial or financial relationships that could be construed as a potential conflict of interest.

## Supplementary Material

Supplementary Movie 1 Control oocytes injected with CCNB1-GFP mRNAs were incubated for 2 h and released for live-cell imaging. We kept the experiments until GVBD. CCNB1 was visualized with GFP. Scale bar, 20 μm.

Supplementary Movie 2 FBXO34-depleted oocytes injected with CCNB1-GFP mRNAs were incubated for 2 h and released for live-cell imaging. We kept the experiments until GVBD. CCNB1 was visualized with GFP. Scale bar, 20 μm.

Supplementary Movie 3 Control oocytes injected with MAP4-eGFP and H2B-mCherry mRNAs were incubated for 4 h and released for live-cell imaging. The time spanning of the movie was from GVBD to MII stage (about 10 h). The spindle was labeled with MAP4-eGFP, and the chromosomes were labeled with H2B-mCherry. Scale bar, 20 μm.

Supplementary Movie 4 FBXO34-overexpressed oocytes injected with MAP4-eGFP and H2B-mCherry mRNAs were incubated for 4 h and released for live-cell imaging. The time spanning of the movie was similar to that of the control group. The spindle was labeled with MAP4-eGFP, and the chromosomes were labeled with H2B-mCherry. Scale bar, 20 μm.
